# Neuromodulation by electrical stimulation timing during picture naming task based on high gamma dynamics

**DOI:** 10.1016/j.ebr.2026.100883

**Published:** 2026-06-25

**Authors:** Kyousuke Kamada, Christoph Kapeller, Mostafa Mohammadpour, Johannes Gruenwald, Christoph Guger

**Affiliations:** a Chitose City Hospital, Japan; b g.tec medical engineering GmbH, Sierningstrasse 14, 4521 Schiedlberg, Austria

**Keywords:** Brain Mapping, ECS, ECoG, High gamma, Language, Neuromodulation

## Abstract

**Objective:**

Routine electrical cortical stimulation (ECS) with a duration of 4 to 6 s evoked various symptoms despite the risks of seizure. Distribution of broadband High Gamma Activity (HGA) in electrocorticography (ECoG) appeared with stable and repeatable results compared to ECS-symptoms. We hypothesized that the symptomatic variation might be caused by ECS timing. This study aims to apply short-term ECS with different timing, taking HGA dynamics into account, and the crucial timing of short-ECS might potentially disrupt functional brain rhythms, causing neuromodulation. The purpose of this study is to identify the results of dynamics by short-term ECS that would shed light neurophysiological basics of routine ECS. This study aims to determine the most effective short-term ECS timing based on HGA dynamics.

**Methods:**

Two patients participated in this study, starting with overt picture-naming (PN), a sham task without ECS, to observe HGA profiles. The ECoG locations with strong HGA were selected for ECS trials with different onset timings (350–1000 ms post-stimulus).

**Results:**

In the PN task with no ECS, HGA peaked between 454 and 684 ms in the patients. ECS before the HGA peaks caused symptoms, and error rates became 92% and 47%, whereas post-peak ECS errors reduced to 37% and 13%. In addition, the response times became significantly slower for pre- vs post-peak ECS.

**Conclusions:**

A strong relationship exists between the HGA and the ECS-timing. Short ECS disrupts brain functions and networks when appropriately aligned with the HGA. Combining HGA dynamics with ECS reveals the functional distribution and physiological dynamics during naming tasks.

**Significance:**

Optimizing the ECS timing can enhance the efficiency and consistency of routine mapping procedures and neuromodulation.

## Highlights

• ECS to the frontal language area before HGA peaks most effectively interrupts picture naming (PN).
• ECS before HGA peaks causes increased response delay and errors.
• Integrating HGA dynamics with ECS timing can improve the clinical mapping efficiency.

## 1 Introduction

Surgical treatments of epilepsy often involve subdural grid implantation to find epileptogenic zones and aura related to pathological activities in the brain. Functional brain mapping is crucial to identify and confirm functional distributions and networks in individual brains. In recent years, electrical cortical stimulation (ECS) and time-frequency analysis of event-related electrocorticography (ECoG) have been employed for functional brain mapping. Technical instruction and active clinical use were initially introduced by Ojemann and Lüders [1], [2]. ECS has demonstrated significant clinical relevance and has been considered the gold standard in neurosurgical practice [3], [4]. ECS still needs to maintain stimulus intensity relatively low to minimize seizure risks. However, we frequently had difficulty evoking targeted symptoms since those depend on stimulus intensities, locations, and timing [5], [6]. In fact, language symptoms are often ambiguous, such as delayed responses, slow speech, and paraphasia [7], [8], [9], [10].

Motor and language functions are the most essential functions in human beings. Lüders et al. initially introduced intracranial recordings of somatosensory-evoked cortical fields during surgery, identifying wave deflection origins such as N1 at 20 ms [1], [11], [12]. This technique enabled us to perform functional mapping to identify sensory and motor cortices under general anesthesia [13]. Kamada et al. later employed a superconducting quantum interference device (SQUID) to measure magnetic fields from the human brain and localize the source of somatosensory-evoked magnetic fields (SEF). Furthermore, the group superimposed the SEF source on anatomical three-dimensional (3D) MRI for preoperative functional simulation [14]. This innovation marked the first non-invasive functional mapping in the human brain. Subsequently, SQUID contributed to magnetoencephalography (MEG), which measured more than hundreds of points from the whole brain and rapidly gained popularity for identifying sensory-motor areas. However, the localization of language-related functions faced several mathematical issues in language-related functional mapping.

On the other hand, functional MRI, which detects blood‑oxygen-level dependence (BOLD) demonstrated obviously frontal language regions with relatively simple semantic tasks [15], [16], [17]. Kamada et al. focused on combining MEG and functional MRI for non-invasive language mapping, achieving a 90% success rate to determine language dominance [18]. However, validating key language centers by ECS was difficult and still challenging in previous reports.

Intracranial recording of evoked ECoG has been clinically utilized to identify the semantic brain activities for over 30 years [19], [20], [21]. Plenty of reports showed that language-related ECoG deflections typically appear later than 200 ms after stimulus presentation, with different tasks [22], [23], [24]. Recent advancements in time-frequency analysis and image fusion techniques have been developed in this field [22], [23], [24], [25]. It's likely a burst of activity that is related to either fast excitatory/inhibitory post-synaptic activity or firing of units. The local field potential is all post-synaptic. Broadband high-gamma activity (HGA) between 70 and 170 Hz in ECoG serves as an additional biomarker, reflecting motor and language-related neural activity. Miller et al. observed increased HGA on the motor cortex during hand poses, characterizing the key biomarker as broadband activity. Similarly, bedside HGA mapping in epilepsy patients indicated specific brain regions showing complex semantic activities [26]. Compared to slower frequency components below 40 Hz [27], HGA visualizes critical insights in short timeframes, with no seizure risk like in ECS [28], [29]. Sinai et al. compared ECS with HGA mapping with a picture-naming task and reported a sensitivity of 85% and specificity of 87.7% compared with ECS mapping results. They hypothesized that the results of the two techniques did not perfectly match due to their underlying different bases, such as stimulation and recording brain oscillation [22]. We encountered ambiguous or unclear symptoms even by routine-ECS of the same location and stimulus intensity. To understand the discrepancy more comprehensively, we decided to analyze various symptoms by changing short-term ECS-stimulus timing on each HGA dynamics.

## 2 Subjects and methods

Three patients with intractable epilepsy participated in this study. All underwent subdural grid implantation for epilepsy diagnosis for video-electrocorticography (ECoG) monitoring and functional cortical mapping by electrical cortical stimulation (ECS). ECoG data were recorded with a 192-channel digital electroencephalography system (Neurofax, Nihon Koden, Japan) at a sampling rate of 1200 Hz. Seizure semiology was captured with a combined normal and infrared video camera operating at 60 frames per second. Language laterality was determined by the Wada test (Table 1). This study was approved by the internal review board of Chitose City Hospital (TRB CH 001–2024). Informed consent has been obtained by all three patients.

**Table 1:** Demographic data of 3 patients. LT (left temporal), RT (right temporal), LF (left frontal), RF (right frontal).

ID	Age/Sex	Dominant side	Verbal IQ	Performance IQ	Seizure	Epileptic foci (contact Fig. 2)
1	36/F	Left	58	68	Sudden generalized	LT (27, 28, 32), after RT resection
2	19/F	Left	95	101	Sudden generalized	RF + LF (R 11, 12; L 68, 69)
3	22/F	Bilateral	83	86	Sudden generalized	RF + LF d(R 12, 13; L 70,75)

Task-related ECoG data were saved digitally at a 1200 Hz sampling rate using a 256-channel g.HIamp biosignal amplifier (g.tec medical engineering GmbH, Austria). The recordings were digitized at 24-bit resolution with a 614 kHz high oversampling to enhance the signal-to-noise ratio.

Routine ECS mapping was performed through electrode pairs with stimulus intensities ranging between 3 and 10 mA. Biphasic electrical pulses with 300 μs phase duration were delivered over 4000 ms, suppressing cortical functions. To expedite ECS mapping, a g.Estim PRO stimulator and switching unit (g.tec medical engineering GmbH, Austria) were utilized to select and stimulation electrode pairs. The optimal electrode pairs elicited clear impairment of language-related functions and indicated the eloquent language areas.

### 2.1 Language tasks

The language tasks included spontaneous speech, picture naming, word reading, and listening to Japanese stories. After general language mapping with various language tasks, the overt PN task was deemed the most straightforward and suitable for analyzing HGA dynamics and the ECS-based neuromodulation.

### 2.2 Specific time-window HGA analysis

For analysis of task-related HGA accumulation, we started the overt PN task without ECS to evoke semantic ECoG. During the ECoG recording, a portable monitor connected to the control computer was placed in front of the patients. The distance between the patients and the monitor was 50 cm to clearly show objects to all patients. The real-time processing system g.HIsys (g.tec medical engineering GmbH, Austria) presented colour pictures on the monitor. A photodiode attached to the edge of the monitor to precisely detect the timing of object presentations, sending external 5 V TTL (transistor-transistor logic) triggers to the g.HIamp amplifier. Simultaneously, a webcam (60 fps) and a microphone (8 kHz) recorded each patient's facial expressions, vocalizations, and speech onset. All recorded data were synchronized for accurate alignment of picture presentation, mouth movements, and vocalizations. Pictures were displayed for 300 ms with a 4050–4700 ms inter-stimulus-interval between two pictures. The jitter assured that there was no anticipation bias in the high-gamma activity. A 1000 ms pre-stimulus period was used as a reference period to z-score the signal.

ECoG data were processed in MATLAB 2023a (Mathworks, USA). A common average reference (CAR) was applied to remove common-mode signals, and visually faulty channels were excluded. Accumulation of HGA between 60 and 170 Hz was identified utilizing the z-scored amplitude envelope after Hilbert and square root transformation to approximate Gaussianity. The z-scores of all trials were combined using Stouffer's approach, and the maximum z-score was used as an activation marker for each location [30].

### 2.3 Neuromodulation Study (short-ECS)

**Fig. 1:**
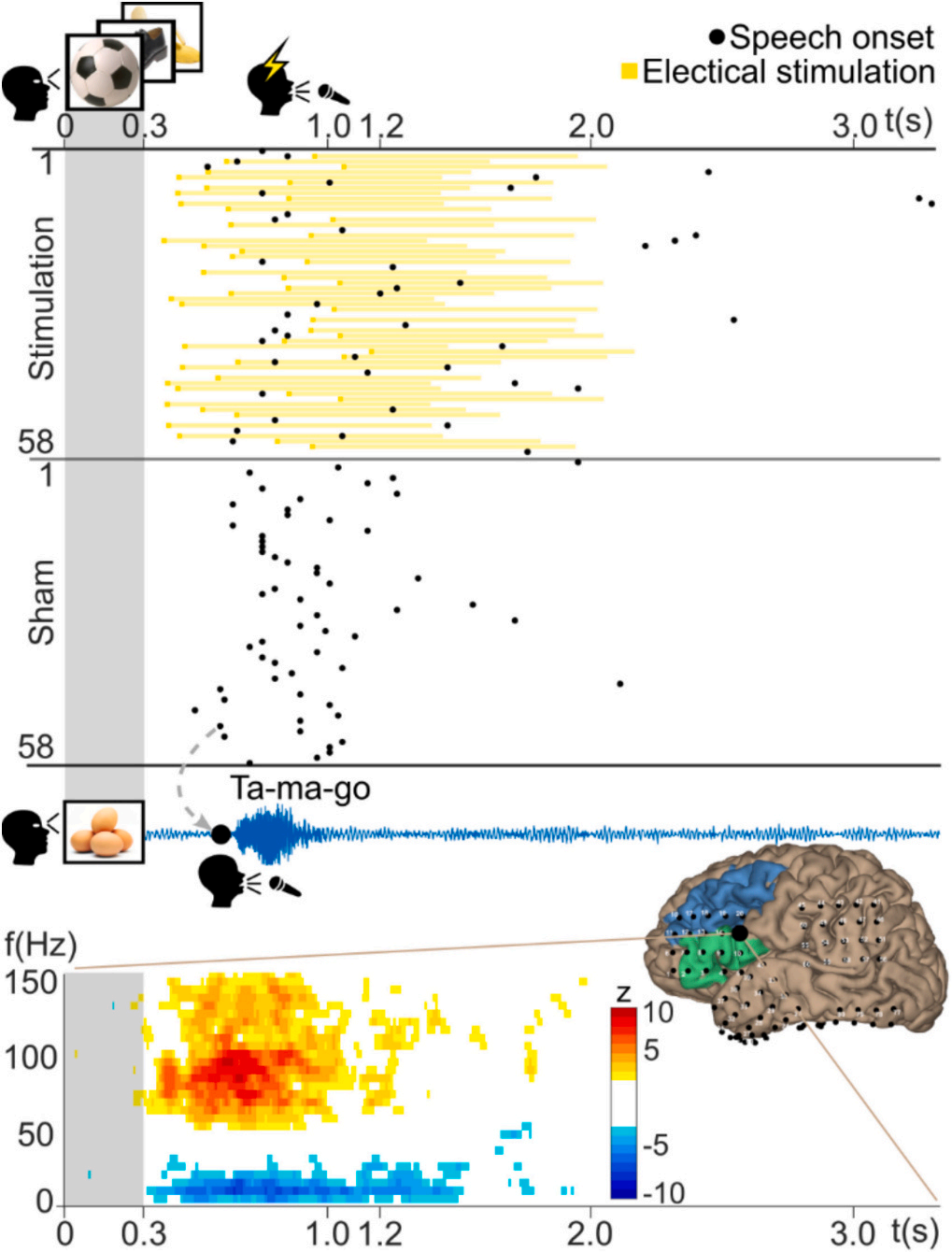
Upper row: Experimental overview of neuromodulation showing black dots are response time and yellow bar is stimulation timing and duration. Response times are shown for 58 stimulation trials and 58 sham trials without stimulation. Lower row: Time-frequency analysis with the strongest HGA accumulation electrode on the left frontal lobe. The z-scores for each trial were calculated based on the 1000 ms pre-stimulus baseline mean and standard deviation of all sham trials and were further combined using Stouffer's approach. (For interpretation of the references to colour in this figure legend, the reader is referred to the web version of this article.)

The neuromodulation part focused on HGA dynamics and comparison with ECS mapping results. We analyzed expressive language functions on regions of interest (ROI), the pars opercularis, triangularis, orbitalis, anterior (area 44, 45), and posterior (area 6) parts of the middle frontal gyrus, since responses of the patients were clear and easy for symptomatic categorization. ECS in those areas mainly causes expressive language errors during the PN tasks in the context of brain surgeries [31], [32]. Stimulated locations with speech disturbance during ECS were considered as language-related areas. The language location with the highest HGA peak was selected as a stimulation target for the short-ECS with stimulus random onsets after the picture presentation (Fig. 1). Thus, stimulation onset varied between 300 and 1100 ms with a train duration of 1000 ms. The stimulation current and phase duration were the same compared to routine ECS.

Responses were categorized as follows:

1. Smooth naming
2. Delayed response (over 2 standard deviations) or hesitation
3. Hesitation
4. Paraphasia
5. Naming mistakes (Paranomia)
6. Speech arrest

Responses 2–6 were defined as “symptoms.”. This classification of symptoms of ECS-facilitated analysis was in relation to HGA dynamics.

## 3 Results

### 3.1 Routine ECS mapping

**Fig. 2:**
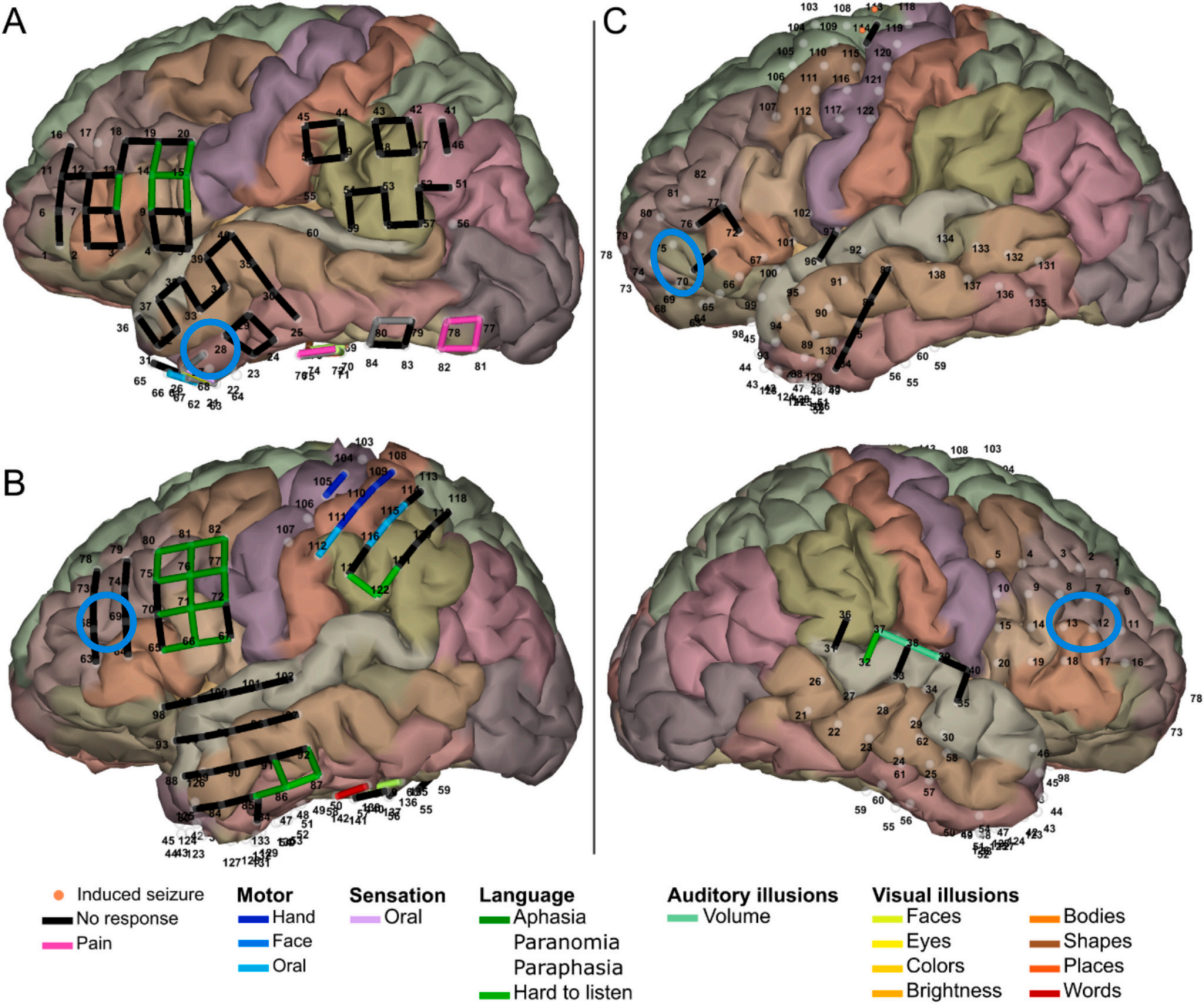
Summary of ECS mapping indicating green lines were electrode pairs, which evoked language impairments. A and B are left hemispheres of Patient 1 and 2, respectively. C shows the left and right hemispheres of Patient 3, who showed 3 green pairs on the right superior temporal gyrus. (For interpretation of the references to colour in this figure legend, the reader is referred to the web version of this article.)

Patient 1: ECS of 5 mA over 4000 ms duration during task presentation impaired language functions on electrode locations 15–20, 14–15, which is on area 6 in the premotor cortex. Pairs of 10–15, 9–14, and 8–13 covered Pars triangularis and opercularis in the inferior frontal gyrus. (Fig. 2A). In particular, ECS with 15–20 and 14–15 (area6) clearly and consistently caused speech arrest.

Patient 2: ECS symptoms by electrode pairs which on the superior part of pars triangularis and opercularis and posterior part of middle frontal gyrus (electrode pairs of 65–66, 66–67, 70–71, 71–72, 72–77, 75–76, 76–77, 75–80, 76–81, 77–82, 76–77, and 81–82 (Fig. 2B). The short-ECS was performed with 7 mA stimulus intensity. In particular, pairs of 77–82 and 76–81 (area 6 and the posterior middle frontal gyrus highly exhibited speech arrest and comprehension difficulties (Fig. 2B).

Patient 3: The ECS on the left hemisphere showed no impairment since the language function was represented bilaterally. However, ECS with 6 mA at the posterior part of right superior temporal gyrus (pair 32–37), together with strong HGA accumulation, induced complex symptoms, including mild paraphasia, very slow responses, difficulties in verbal comprehension, and sound hallucination. Those were outside the ROI and hard to interpret as clear speech impairments (Fig. 2C). Therefore, we skipped Patient 3 from further analysis.

### 3.2 Sham HGA dynamics without ECS

Patient 1 had a relatively low intelligence quotient (IQ), with a task performance of was 83% and a response time of 964 ms, which was acceptable for the neuromodulation study. The performance and response time of Patient 2 were 97% and 752 ms, respectively.

**Fig. 3:**
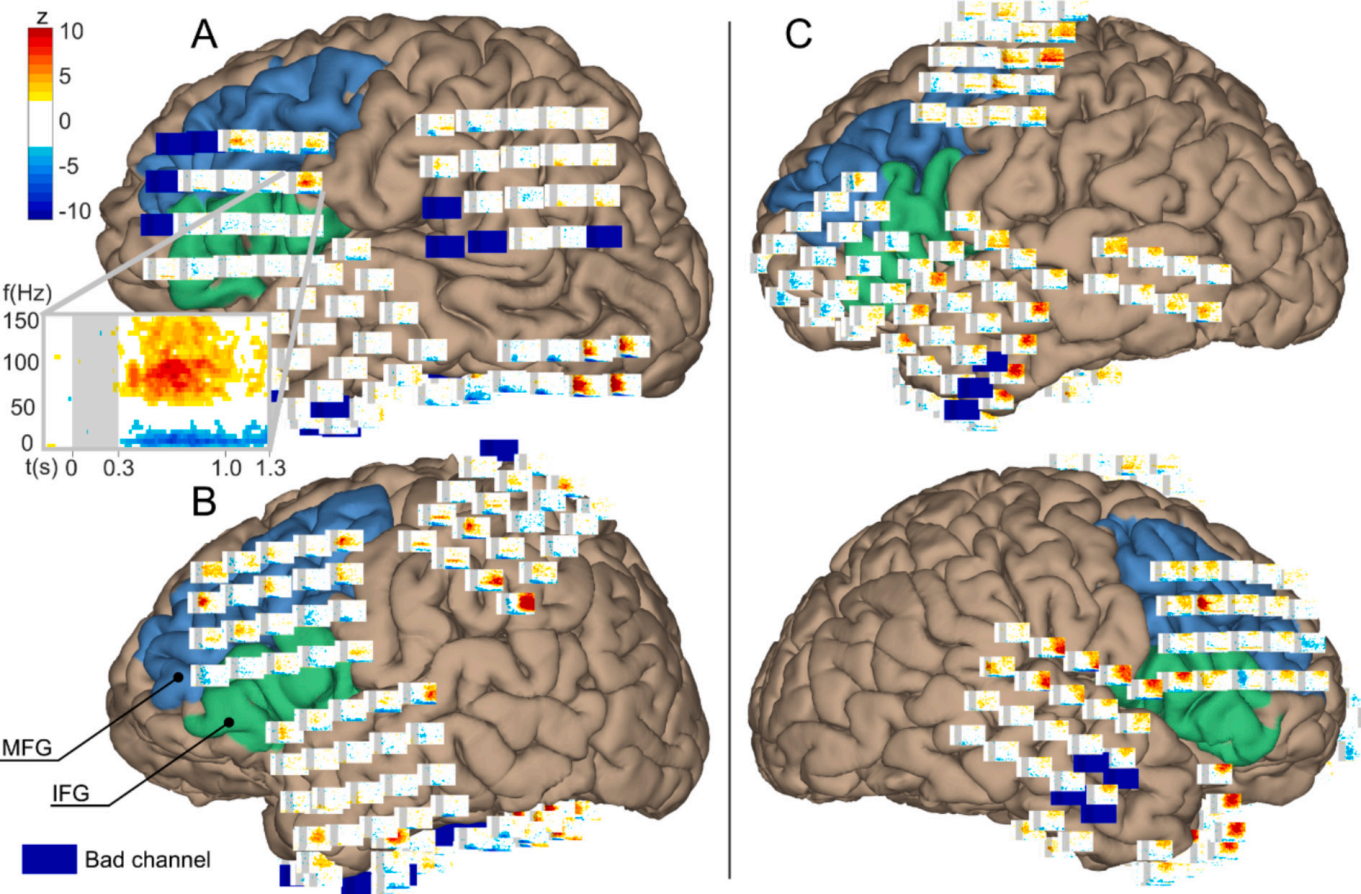
Time-frequency analysis across all channels for three patients. 3D brain models A and B represent Patient 1 and Patient 2, respectively. Model C shows the right and left hemispheres of Patient 3, respectively.

**Fig. 4:**
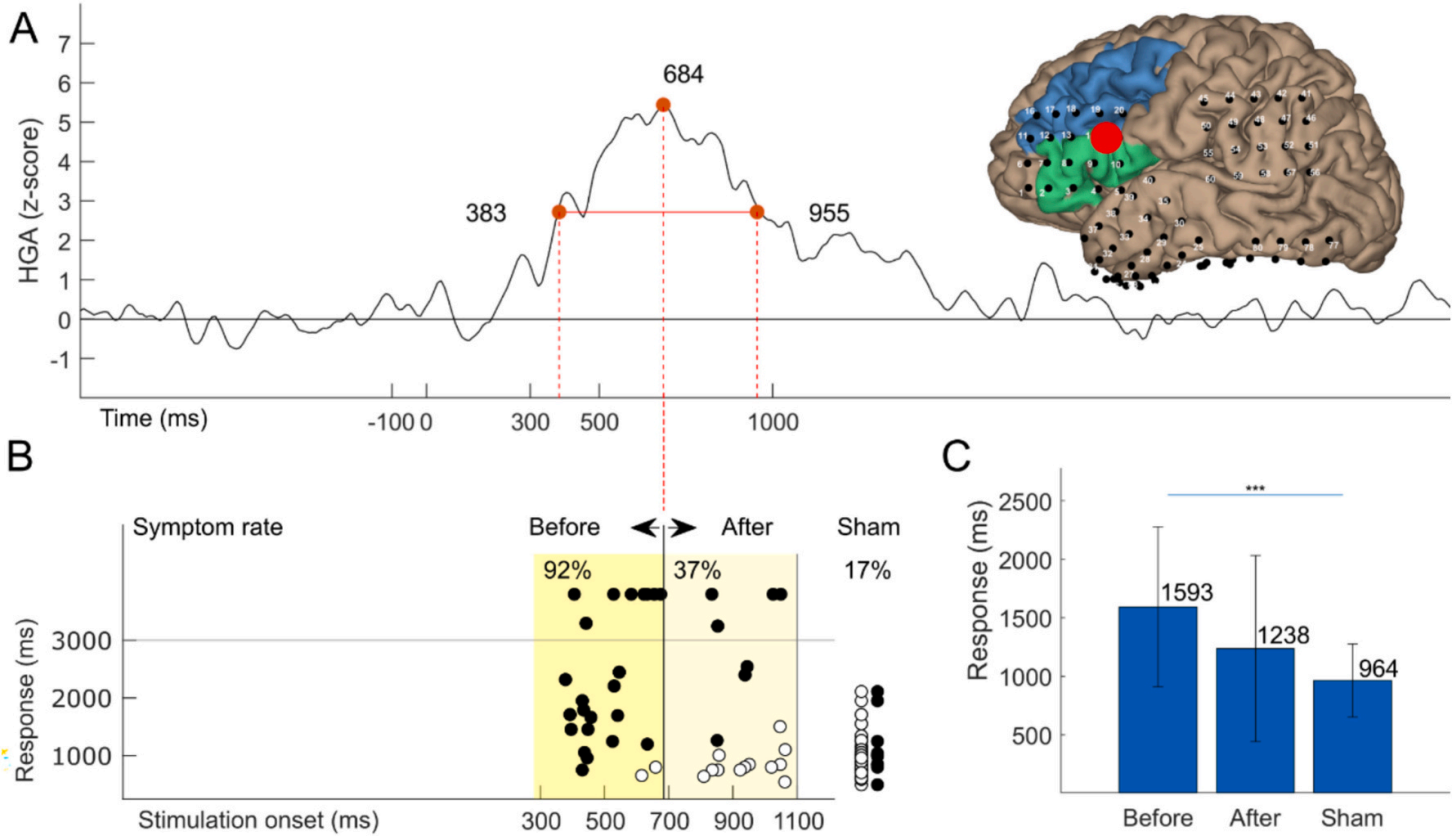
Time-frequency dynamics of Patient 1 peaking at 684 ms on a frontal lobe electrode (A). The error rate was significantly high before the HGA peak. Furthermore, the Sham run demonstrated the lowest error rate (B). The response time of each condition was 1593 ms before the HGA peak, 1238 ms after the peak, and 964 ms, respectively. Error rate and response time are significantly different.

**Fig. 5:**
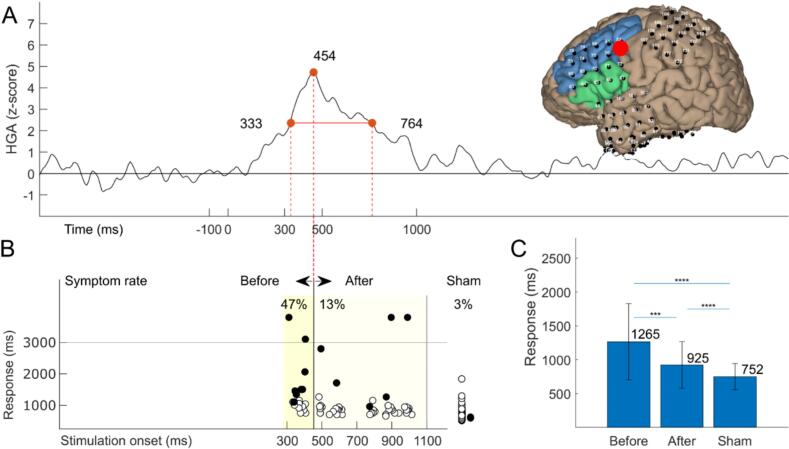
Time-frequency dynamics of Patient 2 peaking at 454 ms on a frontal lobe electrode (A). The error rate was significantly high before the HGA peak. Furthermore, the Sham run demonstrated the lowest error rate (B), as did Patient 1. The response time of each condition was 1265 ms before the HGA peak, 925 ms after the peak, and 752 ms, respectively. Error rate and response time are significantly different.

Fig. 3 shows the time-frequency responses from −200 to 1300 ms, demonstrating the averaged PN-related HGA mainly on the inferior temporal at around 300 ms, and the inferior and middle frontal gyri around 700 ms after the stimulus onset. Early HGA appeared on the lateral aspect of the inferior temporal region in all the patients, which should be related to visual recognition. Compared to routine ECS mapping, the frontal HGA spots were the most important to evoke neuromodulation (Figs. 4A and 5A).

In patient 1, the pair 14 and 15 on the posterior part of the middle frontal gyrus was considered as a key region for neuromodulation (Fig. 3A).

Patient 2 showed the strongest PN-HGA on the middle frontal and supramarginal gyri at 600 ms after the stimulus onset. Electrodes on the supramarginal gyrus and superior temporal lobe showed later HGA peaks (Fig. 3B). Routine ECS on the posterior middle frontal gyrus was the most effective in evoking language impairment (Fig. 3B). We applied short electrical stimulation to the posterior part of the middle frontal gyrus (77 and 82).

Patient 3 exhibited little HGA accumulation on the left frontal and temporal regions, except the inferior temporal gyrus. However, there was obvious bilateral HGA accumulation on electrodes around the right Sylvian fissure. Considering the routine ECS results, HGA map, and Wada test, Patient 3 had bilateral language distribution and was unsuitable for further neuromodulation study.

### 3.3 Neuromodulation results

Despite different HGA peaks in each patient, the peak should be determined depending on the patient's intelligence and language ability. We always used novel pictures for each recording to avoid remembering the presented ones. Therefore, the subjects sometimes hesitated to respond or had difficulty in rapidly naming the similar pictures, e.g., red balloon and red ball, etc. We manually discarded a few responses if the patients reported response troubles during the task after the whole recording. In the sham run, the patients achieved the quickest response time and lowest error rate, which showed statistical differences between groups with ECS and no ECS were assessed using a pairwise single sided Mann-Whitney-*U* Test, followed by Bonferroni correction for multiple tests. As a result, short-term ECS significantly interrupts brain functions, most effectively at around 500 ms to 1000 ms, to temporally block the language network and interrupt the response preparation.

PN-related HGA accumulation of Patient 1 showed the HGA peak at 684 ms after stimulus onset, as shown in Fig. 4A. The ECS trials were separated into “Before” and “After” HGA peak. Black and white dots in Fig. 4B indicate error and correct responses, respectively. After ECS with any timing after stimulus onset, the performance rates were decreased. In a sham measurement with no ECS, her performance rate was 83%. Short ECS, however, before and after 684 ms caused significantly increased error rates, such as 92% and 37%, respectively (Fig. 4C). Latencies with ECS before and after the HGA peaks slowed down to 1238 and 1593 ms, respectively. Thus, the pre-peak ECS response time was significantly slower than the sham latency of 964 ms. The asterisks *** indicate significant differences with a *p*-value <0.001.

Patient 2 quickly understood the task and purpose; her HGA peak and response were earlier with a lower error rate, also with an earlier HGA peak at 454 ms. Fig. 5A exhibits the PN-related HGA over time. Short ECS before and after the HGA peak caused significantly different symptoms. The error rates in Fig. 5B increased from 3% sham to 13% and 47% in ECS with post-HGA and pre-HGA peaks, respectively. Fig. 5C shows the latency of the response in different ECS conditions. The asterisks *** and **** indicate significant differences with a p-value <0.001 and < 0.0001. Short ECS before the HGA peak caused the slowest response time of 1265 ms, significantly slower than after the HGA peak and sham response times.

## 4 Discussion

This study analyzed ECS neuromodulation based on HGA dynamics during overt visual picture naming. It aims to determine the optimal ECS timing during language tasks and frequency-modulated symptoms. This research seeks to enhance our understanding of brain function and improve ECS-neuromodulation strategies for functional brain mapping, comparing with HGA dynamics. As a result, we found that short-ECS before HGA peaks significantly evoked higher errors in semantic tasks than those of the ECS after HGA peaks. In fact, the pre-HGA ECS made response times significantly slower than sham latencies. We suppose the ECS might generally affect the cortical functions.

In the present status, ECS-based functional brain mapping consists mainly of four steps:

1. Determining the appropriate stimulus intensity.
2. Introducing simple and straightforward language tasks, such as PN or word comprehension.
3. Applying ECS with an intensity of less than 10 mA and duration between 4000 and 6000 ms to evoke symptoms, minimizing seizure risks.
4. Careful interpretation of ECS-induced impairments in language-related functions by speech trainers and neurosurgeons.

As mentioned above, shortening and minimizing ECS conditions is crucial to avoid seizure risks. In this study, two patients consistently exhibited speech impairments by the short-ECS with similar stimulus intensity and electrode locations. To explore the best effects of stimulus timing to induce various symptoms, we used a shorter stimulus duration of 1000 ms, with onsets ranging between 300 and 1100 ms after picture presentation. Before the neuromodulation study, patients performed a sham overt PN run without ECS to verify the natural error rate and obtain ECoG time-frequency maps for each electrode location. We recorded HGA dynamics, response times, and symptom rates in sham measurements. Increased HGA in frontal regions indicated critical points for selecting the optimal ECS location. Notably, the interruption of HGA significantly impaired functional language functions. Consequently, the stimulus timing relative to HGA peaks is a crucial factor in functional ECS mapping.

Sinai et al. pointed out that ECS does not yet fully clarify which speech errors are associated with specific brain locations and HGA [22]. They suggested a functional model for more precise and common “location-to-function” language mapping, which might be universally applied to other languages, including Japanese. They, however, introduced the neuroanatomic substrates of word production with covert and overt speech tasks that were variable, depending on the patients. Although consistent HGA accumulation sites revealed exactly the sensory-motor cortices, language tasks engaged the parietal cortex more than typical speech centers.

In the current study, both patients' stimulation sites fall within the “frontocentral language network”—the region surrounding the precentral gyrus where motor, premotor, and inferior frontal language areas interact [33]. Their finding that anarthric errors (motor speech disruption) were most common in the precentral gyrus itself, while speech arrest dominated in the inferior frontal gyrus, explains why our premotor stimulation produced primarily speech arrest rather than articulatory disturbance. The proximity to but not direct overlap with the primary motor cortex suggests disruption of speech initiation and planning networks rather than direct motor execution pathways.

Over the past 20 years, clinical groups have sought additional biomarkers to localize language centers by HGA accumulation. Kunii and Kamada et al. superimposed intracranial electrodes of 12 patients onto a template brain, showing common regions activated during overt picture naming with visual stimuli. Their results demonstrated initial HGA activation in bilateral posterior temporal lobules and fusiform gyri around 250 ms after stimulus onset, and left superior temporal and supramarginal activation after 600 ms. Frontal language activation occurred at approximately 700 ms. This report clearly demonstrated obvious HGA dynamics with overt picture naming and explained why results of routine ECS, MEG, functional MRI and HGA mapping have not been fully matched [29]. As a result, we focused on HGA dynamics and stimulus timing to find the most effective and crucial conditions for functional brain mapping. Recently, several groups paid attention to the relationship between HGA dynamics and functional prognosis [34], [35].

Ogawa and Kamada et al. developed a “real-time” analysis system together with g.tec medical engineering, and compared HGA results from various time windows with ECS mapping [36]. Despite high sensitivity and specificity between 85 and 90% in real-time HGA mapping, which were repeatable, identifying optimal time points remained difficult [37], [38], [39].

In this study, short-ECS was applied before the HGA peak, resulting in the slower response time between 1265 and 1593 ms. After the HGA peak, ECS showed an intermediate response time between 925 and 1238 ms, falling between pre-HGA ECS and sham runs of 752–964 ms in all patients. Notably, the stimulation onset ranged between 300 and 1100 ms after picture presentation. Thus, patient 1 and 2 responded symptom-free before the stimulation in 13.8% and 20.8% of the trials. Those trials fall into the group with stimulation onsets starting at 700–1100 ms after picture presentation, which includes 35% of the trials. This is in line with the observation that stimulation after HGA peak appears to have reduced symptom rate compared to stimulation before HGA peak.

We hypothesize that electrical stimulation of the central nervous system may disrupt functional networks, causing slower responses beyond specific language functions. These findings supported our hypothesis that the short-ECS disrupts functional networks in early HGA and delays responses as well.

We infer that the brain prepares for task processing before HGA peaks, whereas after HGA peaks, most processing might be completed. This current study is a neurophysiological report, showing the effect of the short-ECS on cortical regions, and might contribute to improving optimum ECS procedures in future clinical studies.

A limitation of this study is the small number of patients. However, even with only two patients, the findings and tendency related to the short-ECS were quite pronounced and repeatable. But confirmation in more patients is required to prove that this phenomenon can be generalized.

## 5 Conclusion

This study is the first report of coupled HGA and ECS, and we are just opening the door to understanding the relationship between brain activity and the interruption of networks through ECS. This study demonstrates that even the short-ECS interrupts functional networks, with HGA serving as a critical biomarker for identifying key points during overt picture naming. We believe that integrating HGA dynamics with ECS timing supports ECS as the gold standard for functional brain mapping.

## CRediT authorship contribution statement

**Kyousuke Kamada:** Data curation, Conceptualization. **Christoph Kapeller:** Methodology, Formal analysis. **Mostafa Mohammadpour:** Software, Project administration, Methodology. **Johannes Gruenwald:** Validation, Software, Formal analysis. **Christoph Guger:** Supervision, Funding acquisition.

## Ethical statements

Written informed consent was obtained from the patient for the surgical procedure, the implantation of the neuromodulation device, and the subsequent publication of this case report, including any relevant clinical data and neurophysiology. This report was reviewed and approved by the Ethics Committee of Chitose City Hospital: Approved No. 2025-01.

## Funding sources

Costs of the research were covered by Megumino Hospital, Chitose City Hospital and g.tec medical engineering GmbH.

## Declaration of competing interest

The authors declare the following financial interests/personal relationships which may be considered as potential competing interests: g.tec medical engineering GmbH is a medical device company and manufacturer of equipment in this study. Christoph Guger is CEO of g.tec medical engineering GbmH. Christoph Kapeller, Johannes Gruenwald, and Mostafa Mohammadpour are employees of and receive a salary from g.tec medical engineering GmbH.
